# Light dependent synthesis of a nucleotide second messenger controls the motility of a spirochete bacterium

**DOI:** 10.1038/s41598-022-10556-7

**Published:** 2022-04-26

**Authors:** Jun Xu, Nobuo Koizumi, Yusuke V. Morimoto, Ryo Ozuru, Toshiyuki Masuzawa, Shuichi Nakamura

**Affiliations:** 1 Department of Animal Microbiology, Graduate School of Agricultural Science, Tohoku University, Sendai, Miyagi Japan; 2 Department of Bacteriology I, National Institute of Infectious Diseases, Toyama, Shinjuku-ku, Tokyo Japan; 3 Faculty of Computer Science and Systems Engineering, Kyushu Institute of Technology, Kawazu, Iizuka, Fukuoka Japan; 4 Japan Science and Technology Agency, PRESTO, Honcho, Kawaguchi, Saitama Japan; 5 Faculty of Medicine, Tottori University, Yonago, Tottori Japan; 6 Faculty of Pharmacy, Chiba Institute of Science, Choshi, Chiba Japan; 7 Department of Applied Physics, Graduate School of Engineering, Tohoku University, 6-6-05 Aoba, Aoba-ku, Sendai, Miyagi 980-8579 Japan; 8 Present Address: Department of Bacteriology, Graduate School of Medicine, University of the Ryukyus, Uehara, Nishiharacho, Okinawa Japan; 9 Present Address: Department of Microbiology and Immunology, Faculty of Medicine, Fukuoka University, Fukuoka, Japan

**Keywords:** Microbiology, Bacteriology

## Abstract

Nucleotide second messengers are universally crucial factors for the signal transduction of various organisms. In prokaryotes, cyclic nucleotide messengers are involved in the bacterial life cycle and in functions such as virulence and biofilm formation, mainly via gene regulation. Here, we show that the swimming motility of the soil bacterium *Leptospira kobayashii* is rapidly modulated by light stimulation. Analysis of a loss-of-photoresponsivity mutant obtained by transposon random mutagenesis identified the novel sensory gene, and its expression in *Escherichia coli* through codon optimization elucidated the light-dependent synthesis of cyclic adenosine monophosphate (cAMP). GFP labeling showed the localization of the photoresponsive enzyme at the cell poles where flagellar motors reside. These findings suggest a new role for cAMP in rapidly controlling the flagella-dependent motility of *Leptospira* and highlight the global distribution of the newly discovered photoactivated cyclase among diverse microbial species.

## Introduction

In all domains of life, the nucleotide messengers cAMP, cGMP, c-di-AMP, and c-di-GMP have vital roles in signaling networks, allowing organisms to recognize changes in environmental factors and regulate their physiologies and behaviors. Many cyclic nucleotide-binding proteins have been identified, e.g., the binding of cAMP or cGMP to cyclic nucleotide-gated channels triggers cation flux^1^. The capacitation of mammalian sperm involves phosphorylation of the associated proteins by the cAMP-dependent activation of protein kinase A, and flagellar beating is accelerated by cAMP derived from soluble adenylyl cyclase^2^. Additionally, the c-di-GMP level is known to be involved in the biofilm formation, motility, and virulence of bacteria^3,4^.

Here, we show that the motility of a species of the genus *Leptospira*, a member of the spirochetes, is rapidly modulated by cAMP generated upon light exposure. Motile bacteria possess varied machinery for moving in liquid or over surfaces^5^. A major motility form is flagella-dependent swimming: the peritrichous bacteria *Escherichia coli* and *Salmonella enterica* swim by rotating bundled flagella, and *Vibrio cholerae* and *Pseudomonas aeruginosa* are propelled by a single polar flagellum^6^. While these species possess flagella at the cell exterior, spirochetes such as *Borrelia burgdorferi* (Lyme disease pathogen) and *Treponema pallidum* (syphilis) have their flagella (endoflagella) within the periplasmic space^7^. The endoflagella of spirochetes are thought to rotate within the periplasmic space, rolling or transforming the spiral cell body to generate thrust^7^. We discovered that *Leptospira kobayashii* isolated from soil in Japan^8,9^ drastically alters its swimming pattern immediately after sensing light. We demonstrated the responsibility of cAMP synthesized by a novel photoresponsive adenylyl cyclase for motility control. cAMP affects gene regulation for pili synthesis in cyanobacteria^10–12^, but the soil bacteria respond to light exposure in a subsecond timeframe.

## Results

### Light modulates swimming of soil bacteria

**Figure 1:**
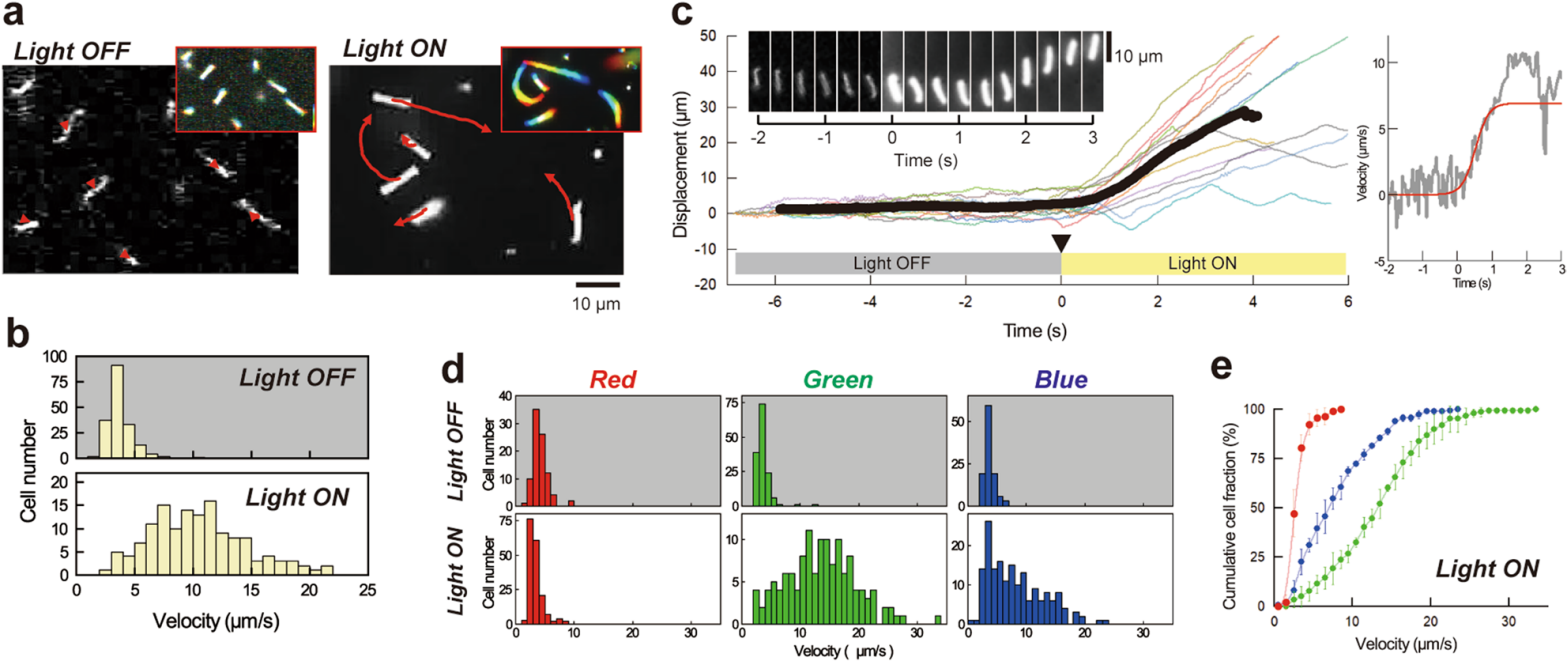
Light stimulation induces unidirectional swimming of spirochetal bacteria. **(a)** Swimming trajectories of *Leptospira kobayashii* cells under low-intensity (termed as “Light OFF” in this study) and high-intensity (termed as “Light ON”) illumination. The results of 2-s tracking are shown. Colored footprints (inset, time courses in the order from red to blue) show almost zero net migration in Light OFF. Note that the bacteria retain vigorous motility in Light OFF (see the scene “Light OFF” in Movie 1). As a light source, a halogen lamp was used with a wide band-pass filter (ca. 400–700 nm). **(b)** Velocities of individual cells measured in Light OFF (n = 189 cells) and in Light ON (n = 150 cells). The data were obtained by three independent experiments. **(c)** Time courses of cell displacement. Example traces obtained from 14 cells (thin colored lines) and the averaged trace (thick black line) are shown. Neutral density filters were removed at the time point (indicated by the triangle and set as 0 s) so that the cells were suddenly exposed to stronger light. The sequential micrographs (inset) show the light-dependent initiation of directional swimming. The right panel shows the time course of velocity (gray) obtained from the mean displacement and the result of sigmoidal curve fitting (red). **(d)** Color dependence of the light-controlled motility. Band-pass filters with center wavelengths of 650 nm, 550 nm, and 488 nm were used for red, green, and blue illumination, respectively. The experiments were repeated three times, and ca. 100 cells were measured in total in each condition. **(e)** Cumulative cell fractions calculated from the histograms of Light ON shown in (**d**). The colors indicate those used for illumination: average values (closed circles) and standard deviations (error bars) of three independent experiments.

The genus *Leptospira* possesses two endoflagella (one flagellum per cell end, Supplementary Fig. 1). *Leptospira* spp. swim by rotating the coiled cell body (swimming mode), and smooth swimming is frequently interrupted by rotation without migration (rotation mode)^13,14^. We found that *L. kobayashii* exclusively showed rotation in the low light condition (0.2 μmol/m^2^/s, “Light OFF” in Fig. 1), but smooth swimming was triggered by light exposure (26.1 μmol/m^2^/s, “Light ON” in Fig. 1, Movie 1, Supplementary Fig. 2). It is known that the transition between the swimming and rotation modes of *Leptospira* spp. is affected by viscosity^15^ and chemical substrates^16,17^, but this is the first report of light-dependent modulation of *Leptospira* motility. The frequency of swimming reversal decreased with light intensity, increasing the migration distance per unit time (Supplementary Fig. 3). Therefore, light-dependent motility was quantified using the swimming velocity of individual cells (i.e., migration distance per second), showing an increase in velocity up to fourfold under light (Fig. 1b, Supplementary Fig. 3). Smooth swimming reached the maximum velocity at ~ 1 s after stimulation (Fig. 1c). The light-responsivity depends on the light intensity, and the duration of unidirectional swimming increased with increasing light intensity (Supplementary Fig. 3). The response to light was observed even at ~ 1 μmol/m^2^/s, which is less than 1/10 of the light in a conventional experimental room illuminated by 32 W fluorescent lamps (500–600 lx) (Supplementary Fig. 3). The bacteria can respond to green and blue light but not red light (Fig. 1d). Cumulative cell fractions obtained from the velocity histograms show that green light induces more cells to swim smoothly than blue light (Fig. 1e).

### Identification of a photoresponsive gene

**Figure 2:**
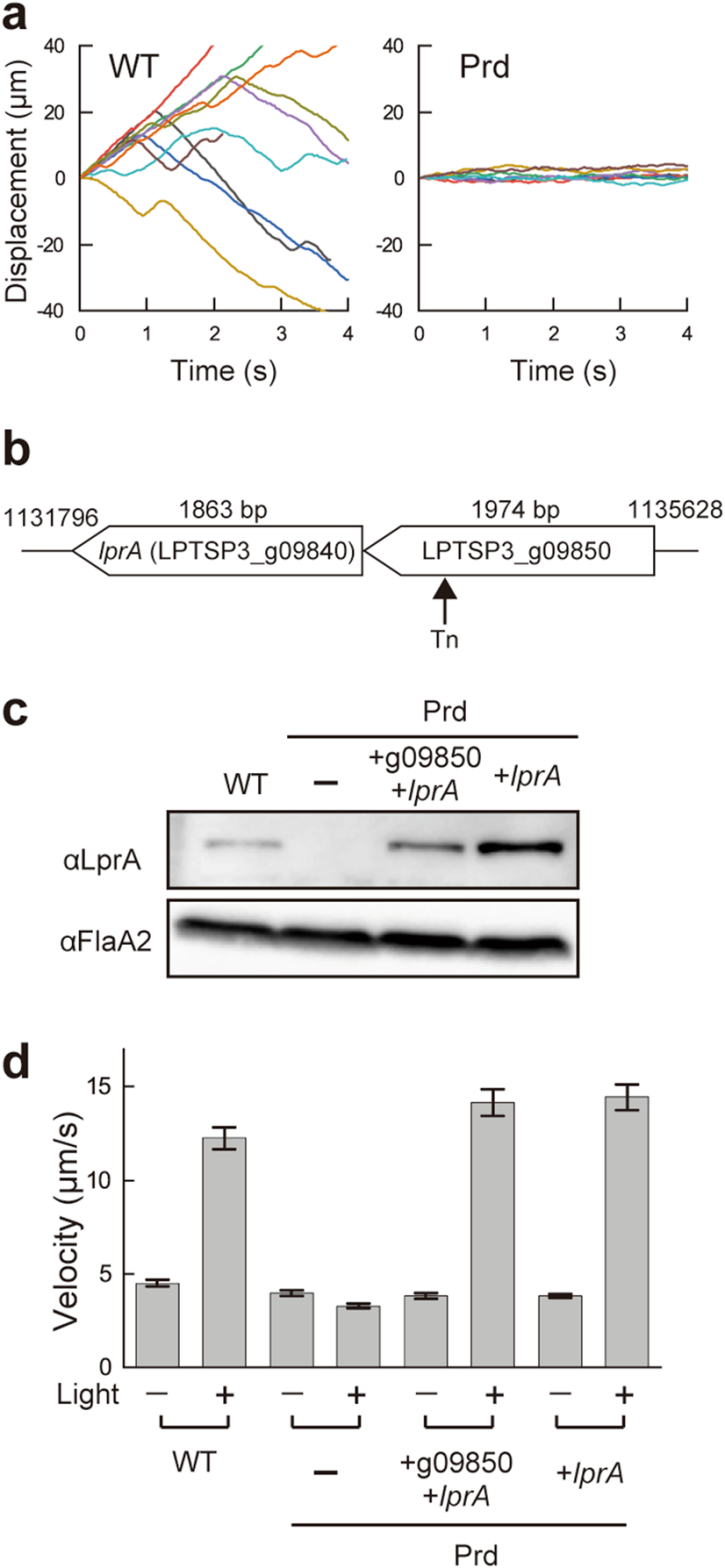
Identification of a photoresponsive gene in the spirochete *Leptospira*. (**a**) Time traces of the cell displacement under light exposure. The traces of 8 cells are shown in each strain. (**b**) Transposon insertion in the photoresponsivity-deficient mutant Prd. The position of the transposon insertion is between 1133919 and 1133920th nucleotides of the *L. kobayashii* chromosome 1 (DDBJ/EMBL/GenBank accession number AP025028). (**c**) Immunoblotting of whole-cell lysates from the wild-type *L. kobayashii*, Prd, and its complemented strains using anti-LprA and anti-FlaA2 (control) antisera. (**d**) Effect of transposon insertion and gene complementation on photoresponsivity. Swimming velocities were measured in Light OFF (−) and Light ON (+) conditions. The average values and standard errors were determined by three independent experiments (ca. 100 cells were measured in total in each condition).

To identify the sensor gene responsible for the *L. kobayashii* photoresponsivity, we explored loss-of-function mutants from a kanamycin-resistant library made by transposon random mutagenesis. One of the ~ 2400 clones retained motility but lacked photoresponsivity (Fig. 2a, Movie 2), and this mutant was named Prd (photoresponsivity-deficient). The Prd mutant carried a transposon insertion in the LPTSP3_g09850 gene (Fig. 2b), but the complementation of the LPTSP3_g09850 gene was not able to restore the photoresponsivity of the mutant (Supplementary Fig. 4). Noting that the LPTSP3_g09840 gene is located immediately downstream of the LPTSP3_g09850 gene and encodes adenylate/guanylate cyclase domain-containing protein, we complemented the Prd mutant with both the LPTSP3_g09850 and LPTSP3_g09840 genes, resulting in the recovery of photoresponsivity (Fig. 2c,d). Then, the photoresponsivity of the Prd mutant was recovered to the wild-type level by complementation of the LPTSP3_g09840 gene (Fig. 2d), indicating that the LPTSP3_g09840 gene is responsible for the photoresponsivity of *L. kobayashii*. We termed the LPTSP3_g09840 gene *lprA* (leptospiral photoresponsive protein A).

### Light-dependent adenylyl cyclase activity of LprA

**Figure 3:**
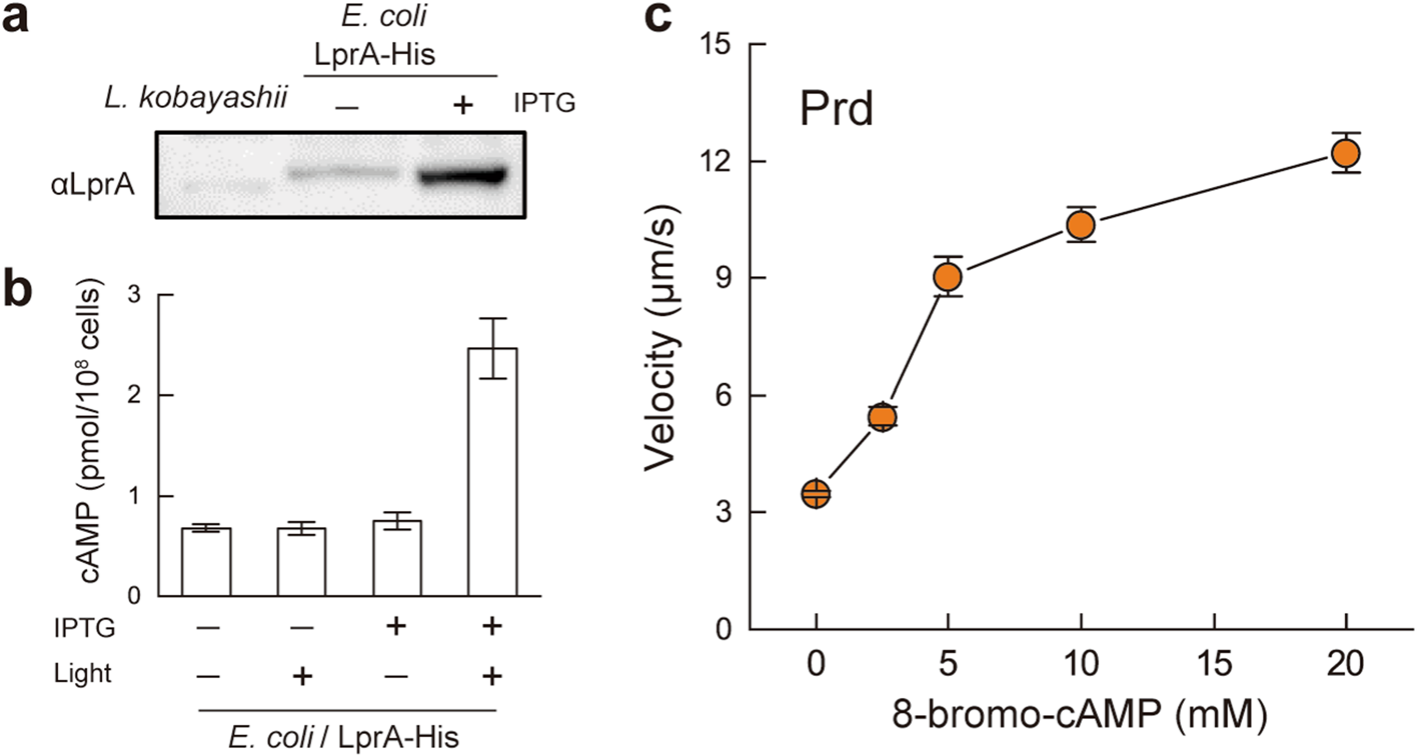
Enzyme activity of LprA. (**a**) Immunoblotting of whole-cell lysates of *L. kobayashii* (1.5 × 10^8^ cells) and *E. coli* expressing LprA-His (2.0 × 10^7^ cells) with anti-LprA antiserum. Since long-time exposure was required for detecting the band of *L. kobayashii*, the *E. coli* sample was diluted at 1:10 for observing the band of the overexpressed proteins clearly. (**b**) Light-dependent synthesis of cAMP in *E. coli* carrying the codon-optimized *lprA*. The average values and standard deviations were determined by three independent experiments. “IPTG − ” and “Light − ” are the uninduced and unilluminated controls, respectively. (**c**) Effect of externally supplemented membrane-permeable cAMP analog (8-bromo-cAMP) on the Prd swimming. Video recording was started less than 1 min after the addition of 8-bromo-cAMP. The average values and standard errors were determined by three independent experiments (ca. 100 cells were measured in total in each concentration).

Photoactivated adenylyl cyclases (PACs) have been found in both eukaryotes and prokaryotes^18–24^. The alignment of LprA with known functional PACs showed that the C-terminal region of LprA contains a domain similar to the AC domain of known PACs, whereas the N-terminal putative sensor domain of LprA was distinct from the conventional PAC sensor BLUF or LOV domain (Supplementary Fig. 5). Since the light-dependent elevation of cAMP concentration was not detected in *L. kobayashii* cells (Supplementary Fig. 6a), we measured the enzyme activity using the *E. coli* overexpression system carrying the codon-optimized *lprA* (Fig. 3a). The overexpressed LprA showed an increase in the cAMP concentration in *E. coli* under light exposure (Fig. 3b). Very little cGMP was detected independently of light, and the small amount could consist of endogenous substrates (Supplementary Fig. 6b). These results suggest that LprA is a novel photoactivation-associated adenylyl cyclase. The Prd mutant cells kept rotating in the Light ON condition, but the addition of a membrane-permeable cAMP (8-bromo-cAMP) induced smooth swimming of the mutant as well as the wild-type cells exposed to light (Fig. 3c). An 8-bromo-cAMP-dependent increase in swimming velocity was also induced in the WT and Prd under the low light condition (0.2 μmol/m^2^/s; Supplementary Fig. 7). These results suggest that the observed modulation of flagellar rotation was induced by cAMP synthesized upon light stimulation.

### Bipolar localization of LprA

**Figure 4:**
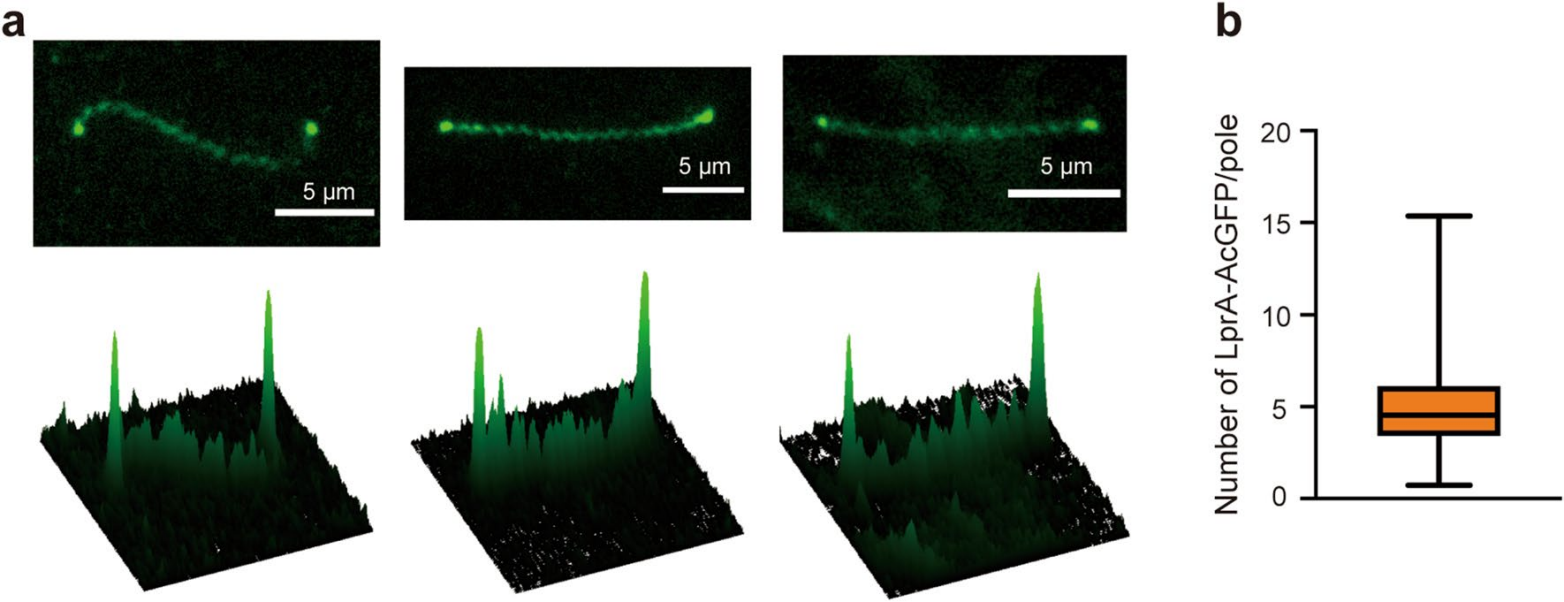
Subcellular localization of LprA. (**a**) Fluorescent images of LprA-AcGFP expressed in the Prd mutant (upper) and 2D intensity profiles (lower). (**b**) Box plot of the number of LprA-AcGFP molecules localized at one end of the cell body. The lower and upper box boundaries are 25th and 75th percentiles, respectively. The line in the middle of the box shows the median number. The lower and upper error lines indicate the smallest and largest values, respectively. 46 cell poles were analyzed.

We examined the localization of LprA in the cell body by labeling the protein with AcGFP1 (green fluorescent protein derived from *Aequorea coerulescens*). AcGFP1 labeling did not affect the photoresponsivity of the bacterium (Supplementary Fig. 8a). Epifluorescence microscopy showed the localization of LprA-AcGFP1 at both ends of the cell body (Fig. 4a). Based on the fluorescence intensity of single AcGFP1 molecules, we estimated that 5.5 ± 3.8 molecules of LprA (n = 46 poles) are localized at one pole (Fig. 4b and Supplementary Fig. 8b), while those in the membrane pool diffuse along the cell body (Movie 3). Although the increase in cAMP concentration in *L. kobayashii* was likely to be below the detection limit (Supplementary Fig. 6), the bipolar localization of LprA could condense cAMP near the flagellar motor, resulting in a rapid response to light.

## Discussion

Organisms react to light through various sensory systems, leading to vision, signaling, and energetic activities. For example, rhodopsins, which are retinal-binding membrane proteins, play a crucial role in vision, ion pumping, and microbial taxis^25,26^, and cryptochromes, which are flavin-binding proteins, are involved in the growth of plants and the circadian clock of animals^27^. Investigating such light-driven proteins deepens our understanding of essential cellular activities. In addition, they have great potential for application as an optogenetic tool, enabling energetic or signaling modulation in live cells under arbitrary spatial and temporal conditions. We showed that *L. kobayashii* modulates its swimming pattern on a subsecond timescale after increasing illumination intensity and that the adenylyl cyclase discovered in this soil spirochete synthesizes cAMP upon light stimulation. The *E. coli* expression system experiment showed that light exposure is indispensable for activating LprA (Fig. 3b), suggesting that LprA functions as a photosensor by itself or is activated by interacting with another photosensor. Since photosensory activity needs a chromophore, the former hypothesis implies that LprA may use a common chromophore with *E. coli*. Although cAMP signaling has been known to be involved in pilus/flagellar biogenesis and biofilm formation in bacteria, these responses are slow and occur through gene regulation^10,12,28^. The current results suggest that cAMP could mediate rapid motility control, although further studies are required to determine whether cAMP acts directly on the flagellar motor.

The genus *Leptospira* comprises pathogenic and saprophytic species^8^. The species that belong to the saprophytic clade S2 containing *L. kobayashii* can respond to light, whereas one of the major pathogenic species, *L. interrogans,* swims even in low light and does not react to light despite carrying the homologous gene (Supplementary Fig. 9). Since the LprA-deficient mutant retains the ability to rotate in one position but cannot migrate by swimming (Movie 2), cAMP-dependent swimming could be important for exploring the environments of free-living species. In contrast, the pathogen motility independent of light is significant for migrating within the host body^29^. Interestingly, a search of the amino acid sequence database showed that many microbial species have a gene homologous to LprA in their genome (Supplementary Fig. 10). Although LprA is not involved in photoresponsive motility in all bacteria, cAMP synthesized by LprA may be used in some signal transductions. Adenylyl cyclases are categorized into six classes, e.g., class I has been found in gamma-proteobacteria, and class II is used by pathogenic bacteria, such as *Bacillus anthracis* and *P. aeruginosa*^30^. Since PACs that have functional similarity to LprA belong to a universal class III used by many species of eukaryotes (e.g., fungi and protozoa) and prokaryotes (e.g., eubacteria and archaea), the successful expression of functional LprA in other bacterial species is the crucial first step for optogenetic application. Understanding the molecular basis of the fast photosensory responses of LprA and cAMP signaling will be a future research topic.

## Methods

### Bacteria and media

*Leptospira* spp. were grown in enriched Ellinghausen-McCullough-Johnson-Harris (EMJH) liquid medium (BD Difco, NJ, USA) at 30 °C for 2 to 4 days (depends on the strains) until the log-phase. *E. coli* strains C41(DE3) and β2163 were grown in L-broth and LB medium supplemented with 0.3 mM diaminopimelate, respectively.

### Screening of a photoresponsivity-deficient mutant by random transposon mutagenesis

Random insertion mutagenesis of *L. kobayashii* strain E30 using *Himar1* transposon was conducted as described previously^31,32^. By conjugation with *E. coli* β2163 carrying pCjTKS2^32^, about 2400 transconjugant colonies were observed on plates of EMJH agar (1% agar) containing kanamycin at a final concentration of 25 μg/ml. Each transconjugant was independently inoculated to 150 μl of liquid EMJH containing kanamycin and grown at 30 °C for 4 days. The grown bacteria were observed using a dark-field microscope (BX50, mercury lamp, 10 × objective, dry condenser; Olympus) for screening photoresponsivity-deficient mutants. The transposon insertion site was identified using the semi-random PCR technique^32^.

For the complementation of photoresponsive-deficient mutant, Prd, the LPTSP3_g09850 and/or *lprA* genes were expressed under the *flgB* promoter^33,34^. The *flgB* promoter region was amplified as previously described^29^, and the LPTSP3_g09850/*lprA* genes, *lprA*, LPTSP3_g09850 gene with FLAG tag, and *lprA* with FLAG tag were amplified from genomic DNA of the *L. kobayashii* E30, and the amplified products were cloned into the *Sal*I-digested pCjSpLe94^35^ by NEBuilder HiFi DNA Assembly cloning (New England BioLabs). For the LprA with GFP joined by a flexible (GGGGS)_3_ linker, *lprA* was amplified as described above, and *gfp* was amplified from pAcGFP1 (Clontech) using the primer containing the (GGGGS)_3_ linker sequence, and the amplified products were cloned into the *Sal*I-digested pCjSpLe94 as described above. Primers used in this study are listed in Supplementary Table 1.

### Immunoblotting experiments

About 1.5 × 10^8^ leptospiral cells suspended in SDS-PAGE sample buffer were subjected to 5–20% SDS-PAGE and Western blotting. The blot was incubated with antisera raised against the peptide fragment of LprA (NH2-LSWADRTDSIYIWK-COOH) and FlaA2^31^ or monoclonal antibody for FLAG tag. Full-size images of immunoblotting data without cropping are shown in Supplementary Fig. 11.

### Motility assay

*Leptospira* culture was diluted 1:20 into a fresh EMJH and was infused into a flow chamber composed of a coverslip (upper side) and a glass slide (bottom side). To examine the effect of membrane-permeable cAMP and cGMP on swimming, 8-bromo-cAMP and 8-bromo-cGMP dissolved in 10 mM Tris–HCl (pH7.0) were added to *L. kobayashii* cells suspended with the same buffer. Motility of leptospires was observed under a dark-field microscope (BX53, Splan 40× , NA 0.75; Olympus, Tokyo, Japan) equipped with a halogen lamp and were recorded by a supersensitive charge-coupled device (CCD) camera (WAT- 910HX, Watec Co., Yamagata, Japan) at a frame rate of 30 frames per second. Swimming trajectories and velocities of individual cells were analyzed using ImageJ software (National Institutes of Health, MD, USA) and VBA-based macros programmed in Microsoft Excel (Microsoft, WA, USA)^14^. Light intensity and wavelength were adjusted with neutral density (ND) filters and bandpass filters with 50-nm bandwidth (FF01-488/50-25 for blue, FF01-550/49-25 for green, FF01-650/54-25 for red; Semrock), respectively. Light intensity was measured using an illuminometer (CHE-LT1, Sanwa Supply INC.).

### Observation of LprA-AcGFP1 subcellular localization

Fluorescence of LprA-AcGFP1 was observed using an inverted fluorescence microscope (IX-83, Olympus) with a 100× oil immersion objective lens (UPLSAPO100XO, NA 1.4, Olympus) and an sCMOS camera (Prime95B, Photometrics). AcGFP1 was excited by a 130 W mercury light source system (U-HGLGPS, Olympus) with a fluorescence mirror unit U-FGFP (Excitation BP 460–480; Emission BP 495–540, Olympus). Fluorescence image processing was performed with the ImageJ version 1.53 software (National Institutes of Health).

The number of LprA-AcGFP1 at the cell pole was estimated by comparing the fluorescence spot intensity at the pole with the fluorescence intensity of a single His-AcGFP1 molecule as previously reported^36^. His-AcGFP1 was purified from *E. coli* C41(DE3) cells carrying pET19b/ His-AcGFP1 using Ni–NTA agarose (Fujifilm Wako). 10 pg/ml of His-AcGFP1 solution was applied to a coverslip washed by 0.1 M KOH and observed by fluorescence microscopy. In the fluorescent images, a rectangular mask for the fluorescent spot of 30 × 30 pixels was applied to the ROI (region of interest). We defined the spot intensity as the sum of all pixel values within the rectangular mask after subtracting the total background intensity from each pixel value. The number of LprA-AcGFP1 per pole was estimated as the intensity of the cell pole divided by the average intensity of a single His-AcGFP1 molecule.

### cAMP ELISA assay

A cAMP ELISA kit (ADI-900-066, Enzo Life Sciences) was used to determine intracellular cAMP levels following the manufacturer’s instructions. *E. coli* C41(DE3) cells carrying pET22b/*lprA*-His were grown in L-broth containing 100 μg/mL ampicillin with or without 1 mM IPTG at 30 °C for 5 h with shaking. The codon usages of the *lprA* sequence were optimized to those of *E. coli* for efficient protein expression. *L. kobayashii* cells were grown at 30 °C for 4 days in EMJH liquid medium until the late-exponential phase. Cells were photo-stimulated with white LED (1156 μmol/m^2^/s) for 3 min and subsequently lysed. Cell lysates were used to calculate cAMP values. The intracellular cAMP concentrations of *E. coli* and *Leptospira* cells were normalized to OD_600_ and OD_420_, respectively.

## Supplementary Information

Supplementary Movie 1.

Supplementary Movie 2.

Supplementary Movie 3.

Supplementary Information 1.

## Data Availability

The data supporting the findings of this study are available from the corresponding author upon request.
