# Supplementary material for: Light dependent synthesis of a nucleotide second messenger controls the motility of a spirochete bacterium
Source: Sci Rep. 2022 Apr 26;12:6825. doi: 10.1038/s41598-022-10556-7 (PMC9043183; doi:10.1038/s41598-022-10556-7)
Supplement: Supplementary file 4 — Supplementary Information 1. [file 41598_2022_10556_MOESM4_ESM.pdf]

Supplementary Information

Manuscript title: Light dependent synthesis of a nucleotide second messenger controls the motility of a spirochete bacterium

Authors: Jun Xu, Nobuo Koizumi, Yusuke V. Morimoto, Ryo Ozuru, Toshiyuki Masuzawa, Shuichi Nakamura

Supplementary Table 1. Primer sequences used in this study.

Supplementary Fig. 1. Morphology of *Leptospira*.

Supplementary Fig. 2. Quantification of the light-dependent change in motility pattern using mean square displacement (MSD) analysis.

Supplementary Fig. 3. Light-intensity dependence of the *L. kobayashii* responsivity.

Supplementary Fig. 4. Effect of *lprA* complementation on the photoresponsivity of the Prd mutant

Supplementary Fig. 5. Sequence homology between LprA and photoactivated adenylyl cyclases

Supplementary Fig. 6. Enzyme activity of LprA

Supplementary Fig. 7. Effect of externally supplemented 8-bromo-cAMP on swimming velocity of the WT and Prd strains under the Light OFF condition (0.2 μmol/m^2/s)

Supplementary Fig. 8. GFP labeling of LprA.

Supplementary Fig. 9. Photoresponsivity of the relative species

Supplementary Fig. 10. Conservation of LprA in prokaryotes.

Supplementary Fig. 11. Full-size images of immunoblotting data.

Movie 1. Photoresponsivity of *L. kobayashii* at 400-750 nm. The cells were observed in low light first (Light OFF), and then exposed to light (Light ON).

Movie 2. Photoresponsivity of Prd mutant. The cells were observed at Light ON condition (see Supplementary Fig. 2)

Movie 3. Prd mutant expressing GFP-labeled LprA. The cell poles are indicated by white arrows. The movie was recorded at 2 frames per second and replayed at 10 times speed.

Supplementary Table 1. Primer sequences used in this study

Target	Primer sequence (5´→ 3´)	
	Forward	Reverse
LPTSP3_ g09850	TAAATGAGGGAGGTTTCCATATGAAAAGATTACATCCGTCAGG	GCGAGGCTGGCCGGCGTCGATCATAATGTTTGAATGGAAACGAAAGCTC
lprA	TAAATGAGGGAGGTTTCCATATGATAGACTTAAATTACATACTCGC	GCGAGGCTGGCCGGCGTCGATTAAGGTTTCCAAATATAAATGGAATCCG
LPTSP3_ g09850/*lprA*	TAAATGAGGGAGGTTTCCATATGAAAAGATTACATCCGTCAGG	GCGAGGCTGGCCGGCGTCGATTAAGGTTTCCAAATATAAATGGAATCCG
LPTSP3_ g09850 with FLAG tag	TAAATGAGGGAGGTTTCCATATGAAAAGATTACATCCGTCAGG	GCGAGGCTGGCCGGCGTCGATCACTTGTCATCGTCATCCTTGTAGT CTAATGTTTGAATGGAAACGA
lprA with FLAG tag	TAAATGAGGGAGGTTTCCATATGATAGACTTAAATTACATACTCGC	GCGAGGCTGGCCGGCGTCGATCACTTGTCATCGTCATCCTTGTAGT CAGGTTTCCAAATATAAATGG
lprA for AcGFP fusion	TAAATGAGGGAGGTTTCCATATGATAGACTTAAATTACATACTCGC	CCGCCGGAACCGCCTCCACCAGGTTTCCAAATATAAATGGAATCCG
AcGFP+(GGGGS)$_3$ linker	GGTGGAGGCGGTTCCGGCGGAGGTGGCTCCGGCGGTGGCG GATCCATGACCATGATTACGCCAAGC	GCTGGCCGGCGTCGATCACTTGTACAGCTCATCCATG

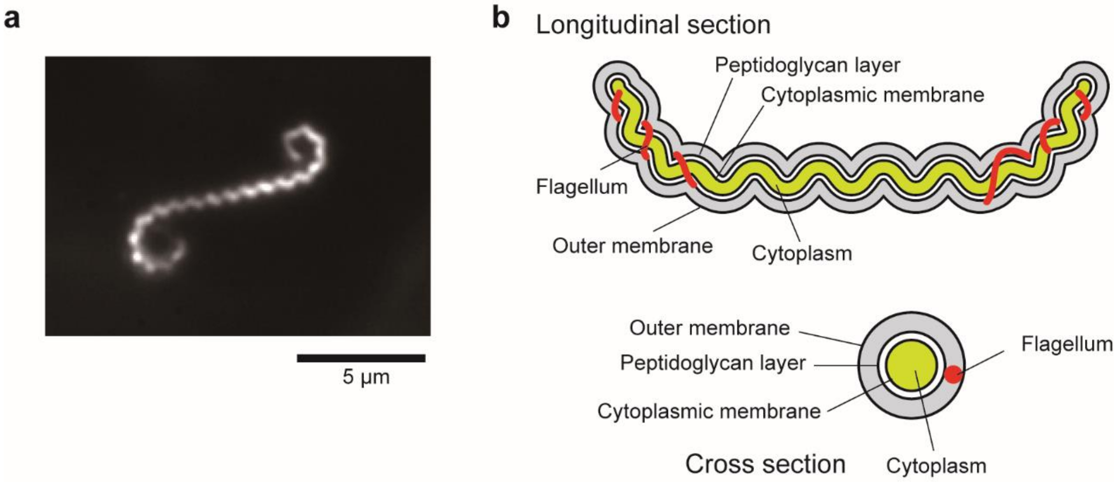

Supplementary Fig. 1. Morphology of *Leptospira*. (a) *Leptospira kobayashii* observed by dark-field microscopy. (b) Schematic diagram depicting the structure of *Leptospira* spp.

Quantification of the light-dependent change in motility pattern using mean square displacement (MSD) analysis.

Consider two particles moving to the right (positive) or left (negative) with time as shown in Supplementary Fig. 2a. The upper particle (gray) moves to both positive and negative directions with the same possibility (*P*), which is so-called free diffusion. In contrast, the movement of the lower one (red) is biased to the positive side. The bead displacements were determined by comparing the assumed probabilities and automatically generated random numbers (rnd); e.g., If P+ > rnd, the bead moves in a positive direction. The result of the simulation is shown in the right panel. The one-dimensional MSD of such particles during Δt is calculated by $MSD(\Delta t) = \langle (x_{i+\Delta t} - x_i)^2 \rangle$, where x_i is the particle position at time i. The time vs MSD plot of the diffusive motion shows linear relation to time, whereas that of the biased movement shows a quadratic curve (Supplementary Fig. 2b, left). Therefore, double-logarithmic plots show linear lines with h slopes of ~1 and ~2 (Supplementary Fig. 2b, right).

We used MSD analysis to quantify the light-dependent motility of *L. kobayashii*. Supplementary Fig. 2c are kymographs of the bacteria moving in dark and bright conditions. The MSD values were computed using data of temporal cell positions, and example traces obtained from about 50 cells are shown in Supplementary Fig. 2d. The double-logarithmic traces and the slopes determined by line fitting to individual traces of all measured samples revealed that most of the bacteria moving in bright have a slope >1, whereas those in the dark have <1 (Supplementary Fig. 2e). These results indicate that the bacterial migration is strongly suppressed while the cell is rotating in the dark, and the movement is instantly directed upon light exposure.

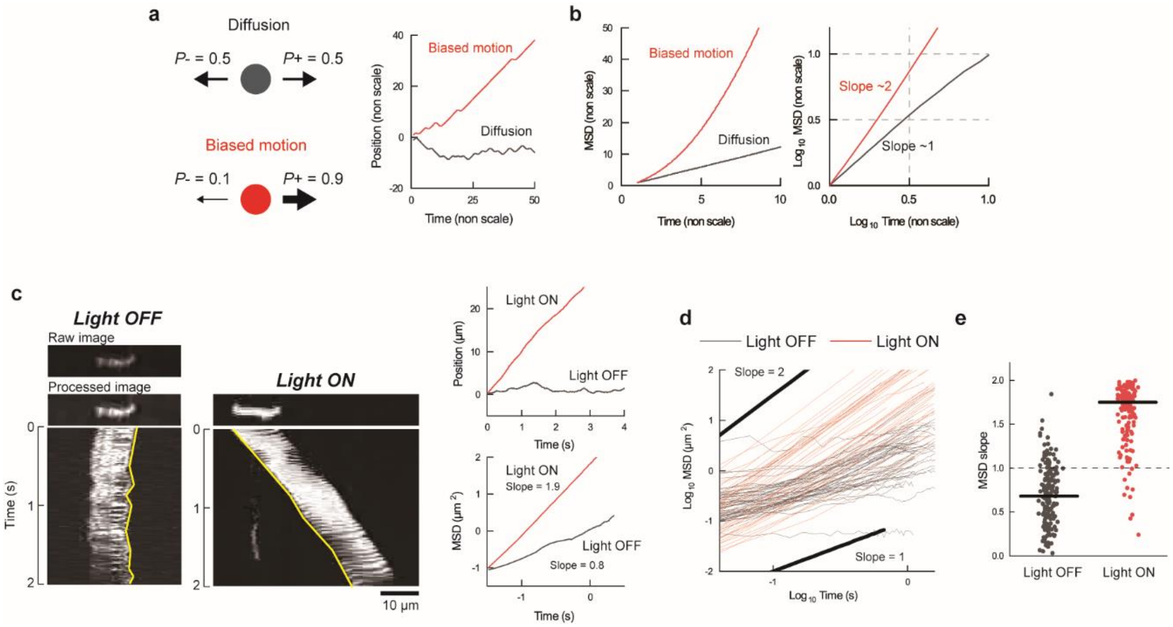

Supplementary Fig. 2. (a) Simulation of diffusive (gray) and biased (red) motions. (b) Time plots of MSD obtained from the simulated bead displacement in ***a***. (c) Kymographs of *L. kobayashii* cells moving in Light OFF and Light ON conditions (see Supplementary Fig. 3). The brightness of video images recorded in the Light OFF condition was adjusted so that the cell position could be determined by ImageJ software. The yellow lines indicate the cell movement. The right panels are time courses of cell positions (upper) and MSD (lower) of the bacteria shown on the left. (d) Example MSD time plots; 59 and 45 traces (cells) are shown for Light OFF and Light ON, respectively. (e) Summary of the values of MSD slope obtained by line fitting to individual paths; 189 and 150 cells were analyzed in Light OFF and Light ON, respectively. The horizontal bars indicate the median: 0.68 for Light OFF and 1.75 for Light ON.

Light-intensity dependence of the *L. kobayashii* responsivity.

We tested the dependence of the light-responsive bacterium *L. kobayashii* on the light intensity. The light illuminance was measured using an illuminometer (CHE-LT1, Sanwa Supply INC.) and converted to photon flux density[1]. Supplementary Fig. 3a-c show that the swimming velocity and MSD slope (see Supplementary Fig. 2) of the bacterial population increase with light intensity. Although the velocity is decreased at 292.8 μmol/m^2/s, the result that the MSD slope is still close to 2 implies uncertain photodamage to the bacteria.

As discussed in Supplementary Fig. 2, time courses of the individual bacterial movements show that light stimulation affects the frequency of back-and-forth motion (Supplementary Fig. 3d). The sequential images recorded at 2.1 μmol/m^2/s show that the bacterium alternates swimming (the periods shown in red in Supplementary Fig.3e) and rotation (those shown in gray), and the rotation period could be interpreted as "tumbling" observed in peritrichous bacteria such as *Escherichia coli*. Exponential distributions were obtained by measuring the time for unidirectional swimming (the left panels of Supplementary Fig.3f), and the rate constants of the transition from swimming to tumbling ($k_{Sw\rightarrow Rot}$) were determined by exponential fitting to the experimental histograms using $exp(-k_{Sw\rightarrow Rot}t)$ (red lines

are the results of fitting). The reversal rate is decreased with increased light intensity (the right panel), indicating that the light stimulation suppresses the reversal event of the flagellar rotation. Swimming time determined as a reciprocal of $k_{Sw\rightarrow Rot}$ is extended with the light intensity (Supplementary Fig 3g), where the swimming time at 0.2 μmol/m^2/s was plotted as zero because of no detectable swimming during observation.

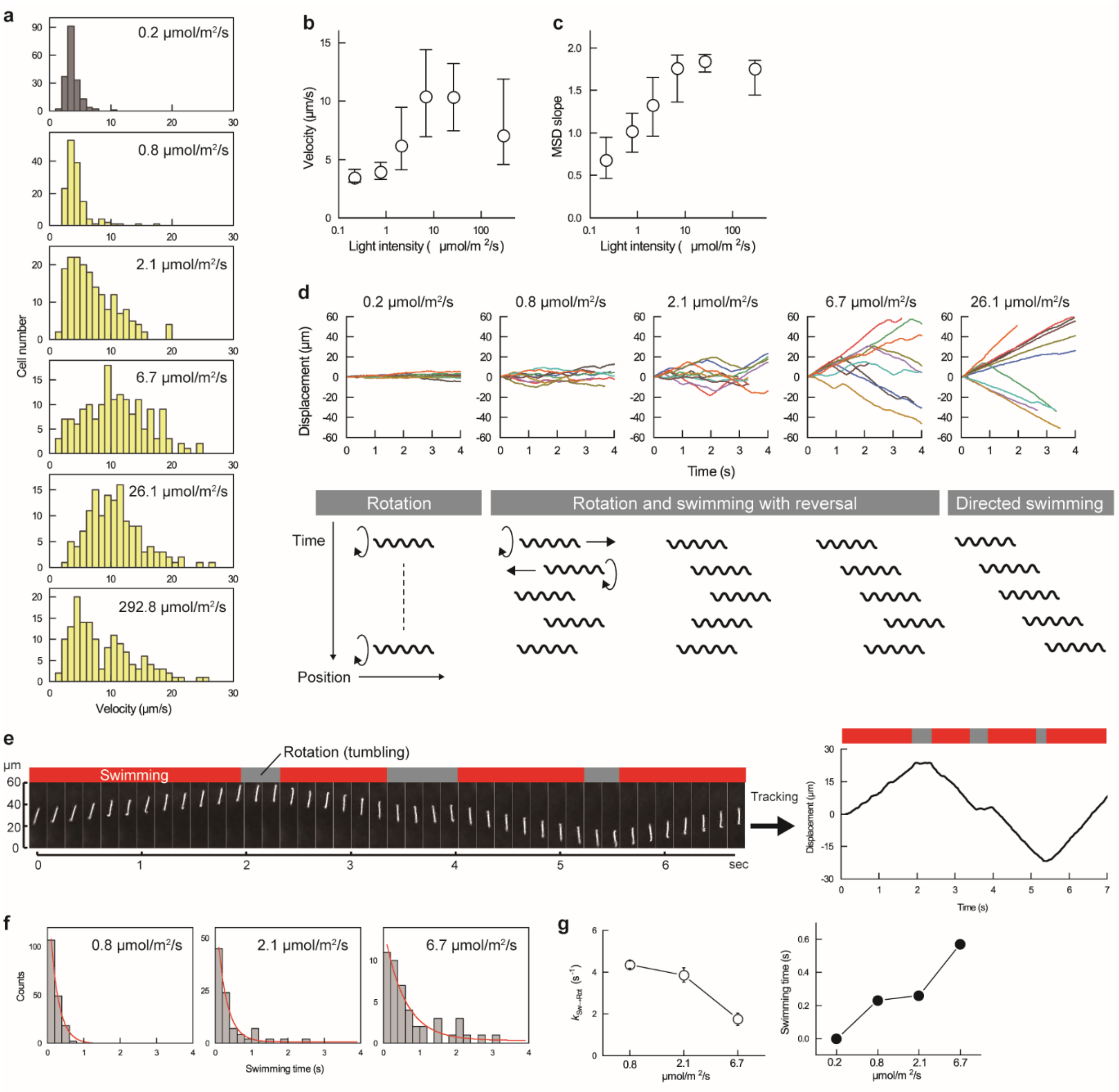

Supplementary Fig. 3. (a) Velocity distributions of *L. kobayashii* swimming in various light-intensity conditions. (b) and (c) show the 25th (lower whiskers), 50th (open circles), and 75th (upper whiskers) percentiles of velocity and MSD slope (see Supplementary Fig 2), respectively. Illuminances indicated in the histograms were average values of five measurements. Each histogram was obtained by three independent experiments, and 183 cells for 0.2 μmol/m^2/s, 145 cells for 0.8 μmol/m^2/s, 179 cells for 2.1 μmol/m^2/s, 165 cells for 6.7 μmol/m^2/s, 141 cells for 26.1 μmol/m^2/s, and 150 cells for 292.8 μmol/m^2/s were measured in total. The data of 0.2 μmol/m^2/s and 26.1 μmol/m^2/s were the same as Light OFF and Light ON in Fig 1. (d) The

upper panels are time courses of bacterial movements. Trajectories obtained from 10 bacteria were shown in each condition. The lower cartoons schematically explain the bacterial movements observed in each light intensity. (e) Sequential images of a *L. kobayashii* cell swimming at the light intensity of 105 lux (left), and the results of cell tracking (right). (f) Distributions of the swimming time. The red curves are the results of the exponential fitting. (g) The rate constants of the transition from swimming to rotation determined by the fitting in ***f*** (left; error bars are the standard error of fitting) and the reciprocals of the rate constants, i.e., the swimming time (right).

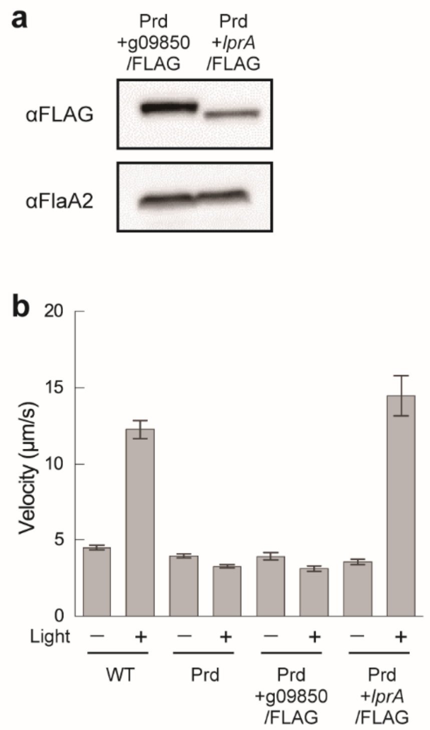

Supplementary Fig. 4. Effect of the *lprA* complementation on the photoresponsivity of the Prd mutant. (a) Immunoblotting of whole-cell lysates from the Prd mutant complemented with the LPTSP3_g09850 gene with FLAG-tag or *lprA* with FLAG-tag. The anti-FlaA2 antiserum and monoclonal antibody for FLAG tag were used. The full-size image without cropping is shown in Supplementary Fig. 11. (b) Swimming velocities measured in Light OFF (−) and Light ON (+) conditions. Average values and standard errors are shown (n= 75 cells for WT-Light OFF, 193 cells for WT-Light ON, 85 cells for Prd-Light OFF, 169 cells for Prd-Light ON, 38 cells for LPTSP3_g09850/FLAG-Light OFF, 70 cells for LPTSP3_g09850/FLAG-Light ON, 45 cells for *lprA*/FLAG-Light OFF, and 39 cells for *lprA*/FLAG-Light ON).

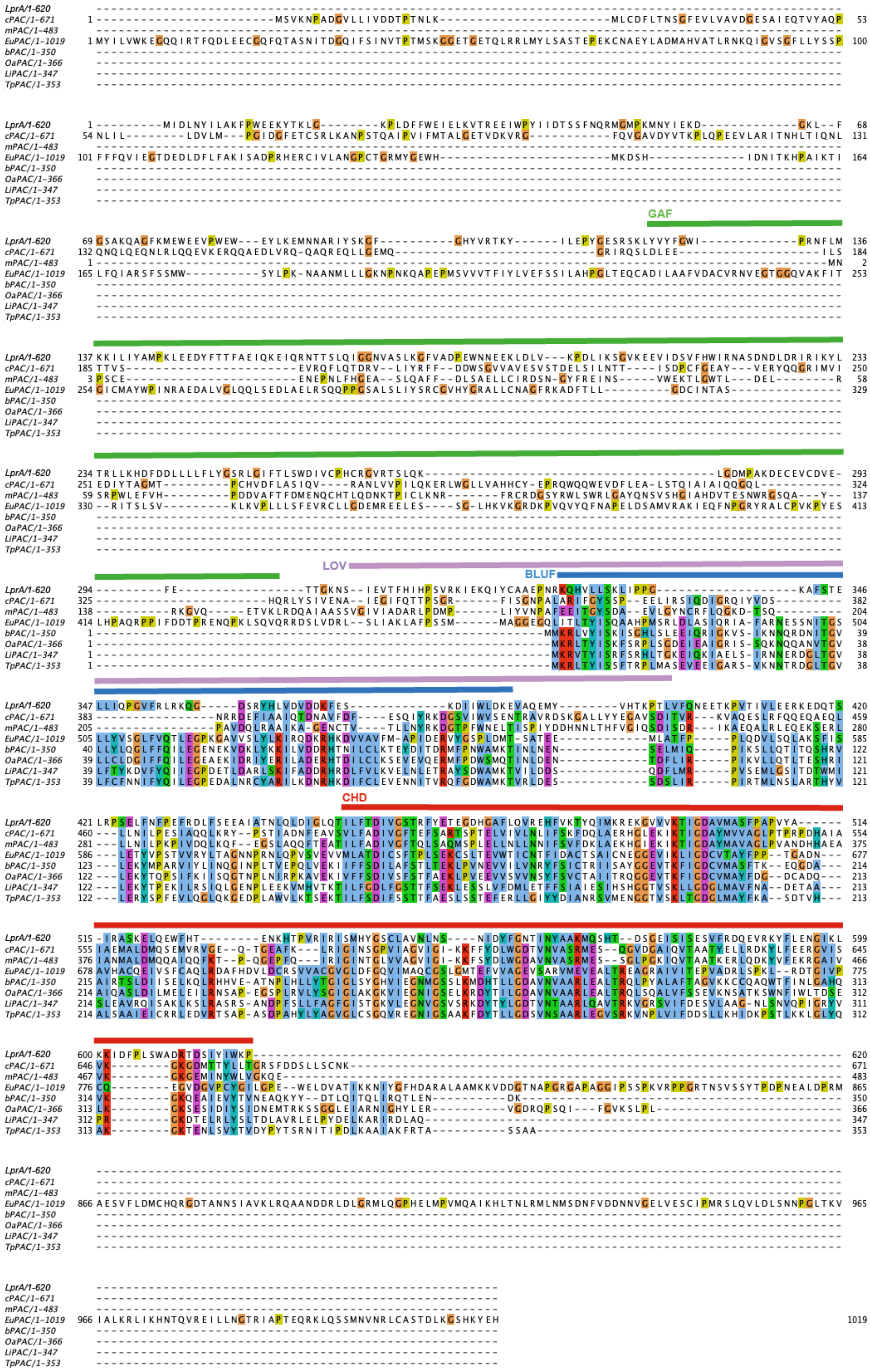
LprA/1-620
cPAC/1-671
mPAC/1-483
EuPAC/1-1019
bPAC/1-350
OaPAC/1-366
LiPAC/1-347
TpPAC/1-353
GAF
LOV
BLUF
CHD

Supplementary Fig. 5. Alignment of amino acid sequences of LprA with known functional PACs. The source organisms are *Microcoleus* sp. *PCC 7113* (cPAC)[2], *Microcoleus chthonoplastes* PCC7420 (mPAC)[3], *Euglena gracis* (EuPAC)[4], *Negleria grubei* (NgPAC2)[5], *Baggiatoa* spp. (bPAC)[6], *Oscillatoria acuminata* (OaPAC)[7], *Leptonema illini* (LiPAC)[8], and *Turneriella parva* (TpPAC)[9]. Multiple sequence alignment was analyzed and visualized using Jalview[10]. Only cPAC contains GAF domain (green bar); only mPAC contains LOV domain (purple bar); EuPAC, bPAC, OaPAC, LiPAC, and TpPAC have BLUF domain (blue bar) as a sensor. LprA contains the cyclase homology domain (CHD, indicated by the red bar) but has neither BLUF nor LOV domain.

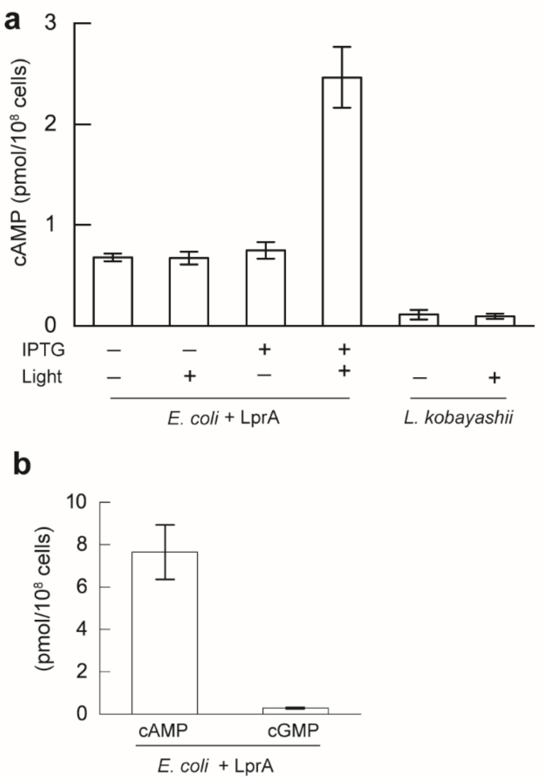

Supplementary Fig. 6. Enzyme activity of LprA. (a) The cAMP concentration measured in the *E. coli* carrying the codon-optimized LprA (*E. coli* + LprA) and *L. kobayashii* with or without light exposure for 3 min. The average values and standard deviations were determined by three independent experiments. "IPTG−" and "Light−" are the uninduced and unilluminated controls, respectively. (b) The cAMP and cGMP concentration measured in *E. coli* + LprA after light exposure for 30 min. Average values and standard deviations of three independent experiments are shown.

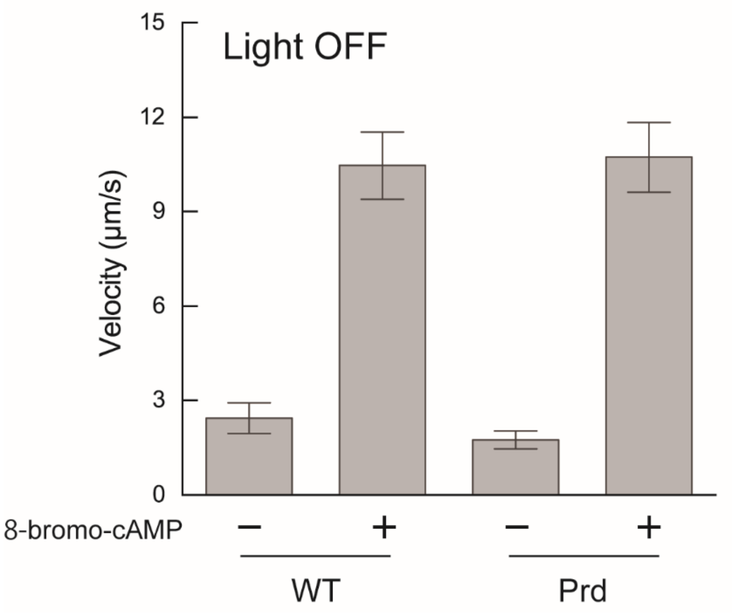

Supplementary Fig. 7. Effect of externally supplemented 8-bromo-cAMP on swimming velocity of the WT and Prd strains under Light OFF condition (0.2 μmol/m^2/s; see Supplementary Fig. 3). The observation was started less than 1 min after the addition of 20 mM 8-bromo-cAMP. The average values and standard errors of 20 cells are shown.

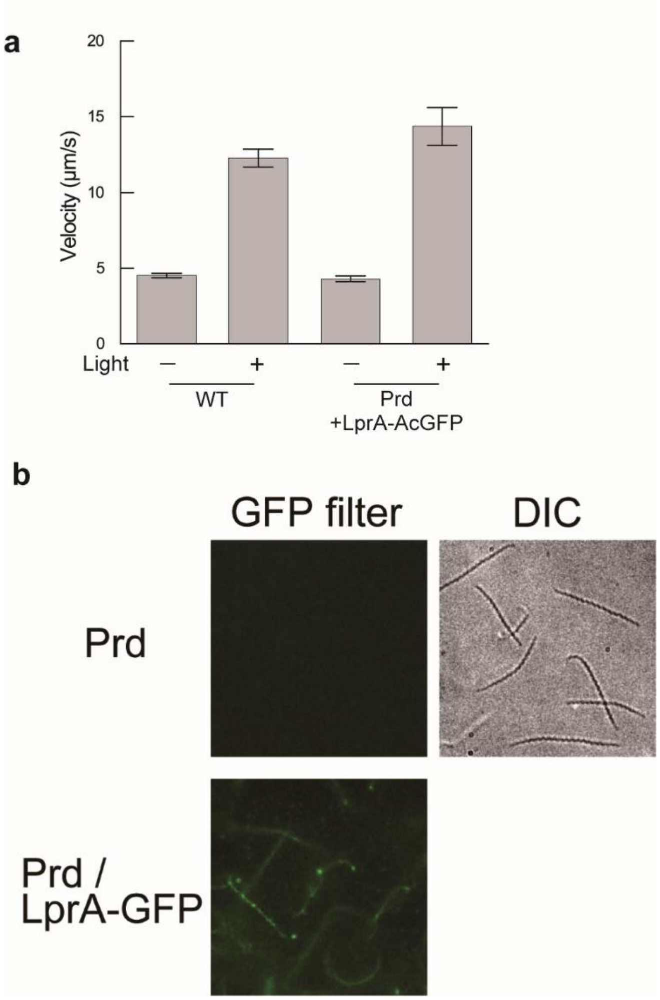

Supplementary Fig. 8. GFP labeling of LprA. (a) Effect of GFP labeling on photoresponsivity of *L. kobayashii.* The data of WT are the same as those shown in Fig 1d (green). For Prd, 46 cells and 55 cells were measured in Light OFF (−) and Light ON (+) with the green filter, respectively. Average values and standard errors are shown. (b) No fluorescence is observed in the Prd strain without carrying the plasmid.

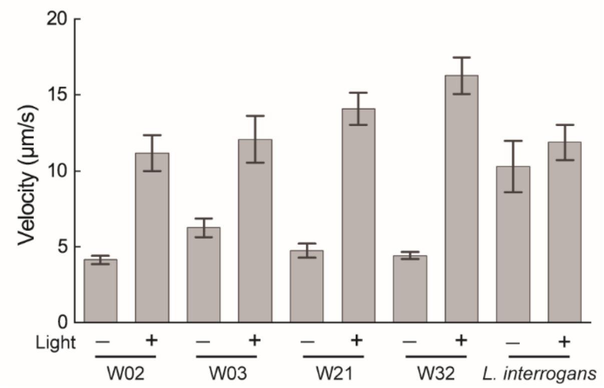

Supplementary Fig. 9. Photoresponsivity of the *Leptospira* species that are phylogenetically related to *L. kobayashii*. The strains were isolated from water and classified into the saprophytic clade including *L. kobayashii* based on the 16S ribosomal RNA sequence[11]. *L. interrogans* is one of the most important pathogenic species, causing the worldwide zoonosis leptospirosis. The swimming velocity was measured in Light OFF (–) and Light ON (+), and average values and standard errors determined from data of 20–50 cells are shown.

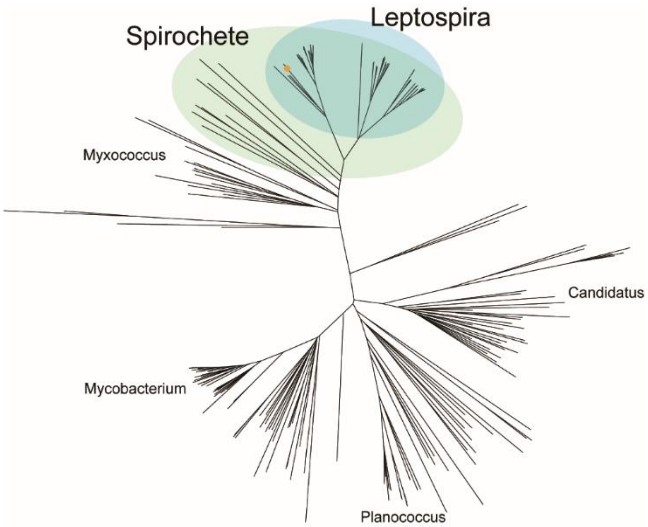

Supplementary Fig. 10. Conservation of LprA in prokaryotes. The orange dot indicates *L. kobayashii*. Homologous genes of LprA were searched by PSI-BLAST program (NCBI). Maximum likelihood phylogenetic trees were constructed and visualized using MEGA X[12] and iTOL program[13].

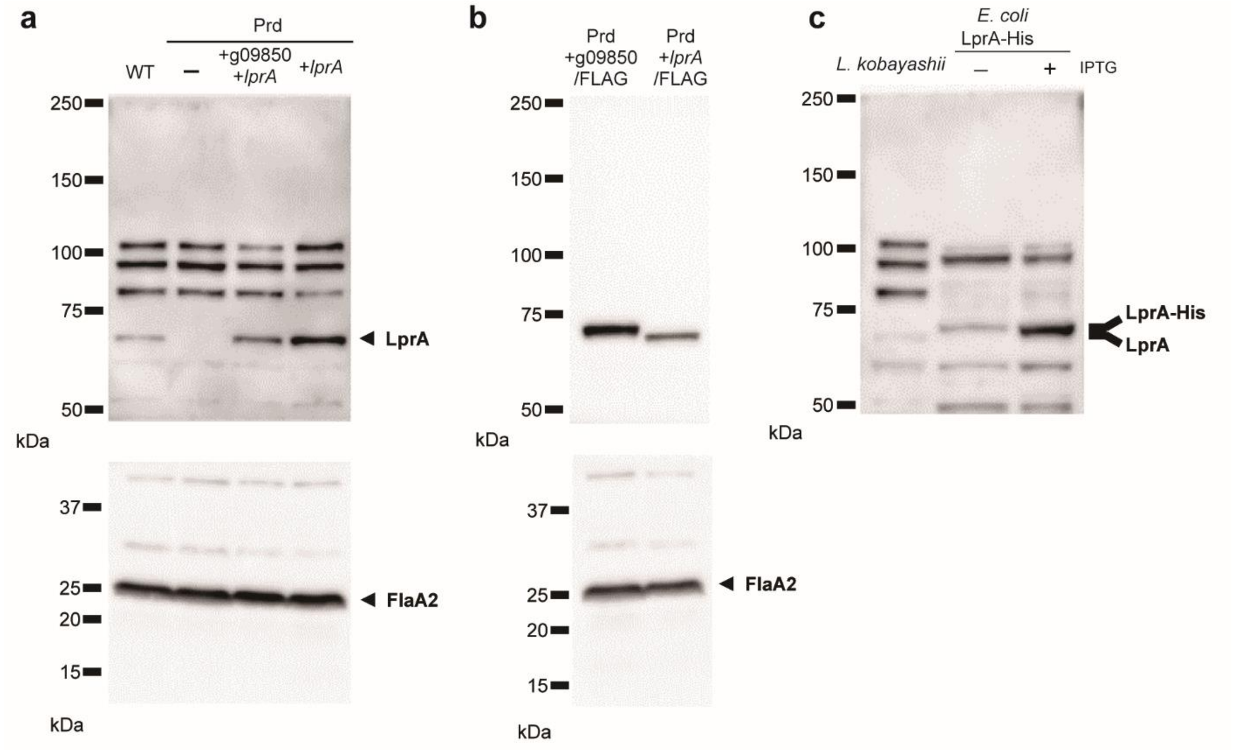

Supplementary Fig. 11. Full-size images of immunoblotting data before cropping. (a) The full-size image of Fig. 2c. The blot was cut into two pieces at approximately 50 kDa and the upper and lower blots were probed with anti-LprA and anti-FlaA2 antiserum, respectively. (b) The full-size image of Supplementary Fig. 4a. The blot was cut into two pieces at approximately 50 kDa and the upper and lower blots were probed with monoclonal antibody for FLAG tag and anti-FlaA2 antiserum, respectively. (c) The full-size image of Fig. 3a. The blot was cut into two pieces at approximately 50 kDa and the upper blot was probed with anti-LprA antiserum.

References

1. Thimijan, R. W. & Heins, R. Photometric, radiometric, and quantum light units of measure: A review of procedures for interconversion. *Horticultural Science* **18**, 818–822 (1983).

2. Blain-Hartung, M. *et al.* Cyanobacteriochrome-based photoswitchable adenylyl cyclases (cPACs) for broad spectrum light regulation of cAMP levels in cells. *J Biol Chem* **293**, 8473–8483 (2018).

3. Raffelberg, S. *et al.* A LOV-domain-mediated blue-light-activated adenylate (adenylyl) cyclase from

the cyanobacterium *Microcoleus chthonoplastes* PCC 7420. *Biochem J* **455**, 359–365 (2013).

4. Iseki, M. *et al.* A blue-light-activated adenylyl cyclase mediates photoavoidance in *Euglena gracilis*. *Nature* **415**, 1047–1051 (2002).
5. Penzkofer, A. *et al.* Photo-dynamics and thermal behavior of the BLUF domain containing adenylate cyclase NgPAC2 from the amoeboflagellate *Naegleria gruberi* NEG-M strain. *Chemical Physics* **412**, 96–108 (2013).
6. Stierl, M. *et al.* Light modulation of cellular cAMP by a small bacterial photoactivated adenylyl cyclase, bPAC, of the soil bacterium *Beggiatoa*. *J Biol Chem* **286**, 1181–1188 (2011).
7. Ohki, M. *et al.* Structural insight into photoactivation of an adenylate cyclase from a photosynthetic cyanobacterium. *PNAS* **113**, 6659–6664 (2016).
8. Penzkofer, A., Tanwar, M., Veetil, S. K. & Kateriya, S. Photo-dynamics of photoactivated adenylyl cyclase LiPAC from the spirochete bacterium *Leptonema illini* strain 3055T. 24.
9. Penzkofer, A., Tanwar, M., Veetil, S. K. & Kateriya, S. Photo-dynamics of photoactivated adenylyl cyclase TpPAC from the spirochete bacterium *Turneriella parva* strain H^T. *J Photochem Photobiol B* **153**, 90–102 (2015).
10. Waterhouse, A. M., Procter, J. B., Martin, D. M. A., Clamp, M. & Barton, G. J. Jalview Version 2—a multiple sequence alignment editor and analysis workbench. *Bioinformatics* **25**, 1189–1191 (2009).

11. Masuzawa, T. *et al.* Comparison of *Leptospira* species isolated from environmental water and soil in Japan. *Microbiology and Immunology* **63**, 469–473 (2019).

12. Kumar, S., Stecher, G., Li, M., Knyaz, C. & Tamura, K. MEGA X: Molecular evolutionary genetics analysis across computing platforms. *Mol Biol Evol* **35**, 1547–1549 (2018).

13. Letunic, I. & Bork, P. Interactive Tree Of Life (iTOL) v5: an online tool for phylogenetic tree display and annotation. *Nucleic Acids Research* (2021) doi:10.1093/nar/gkab301.
